# Serological and Molecular Prevalence of *Babesia caballi* in Apparently Healthy Horses in Israel

**DOI:** 10.3390/pathogens10040445

**Published:** 2021-04-08

**Authors:** Sharon Tirosh-Levy, Monica L. Mazuz, Igor Savitsky, Dana Pinkas, Yuval Gottlieb, Amir Steinman

**Affiliations:** 1 Koret School of Veterinary Medicine, The Hebrew University of Jerusalem, Rehovot 7610001, Israel; danapinkas@gmail.com (D.P.); gottlieb.yuval@mail.huji.ac.il (Y.G.); amirst@savion.huji.ac.il (A.S.); 2 Division of Parasitology, Kimron Veterinary Institute, Beit Dagan 50250, Israel; monicaL@moag.gov.il (M.L.M.); igors@moag.gov.il (I.S.)

**Keywords:** equine piroplasmosis, *Babesia caballi*, IFAT, nested PCR, risk factors

## Abstract

*Babesia caballi* is a tick-borne hemoparasite of equines and one of the causative agents of equine piroplasmosis, which poses a great concern for the equine industry regarding animal welfare and international horse movement. The parasite is endemic in Israel; however, its seroprevalence in the area was never evaluated due to antigenic heterogenicity in the gene used in the commercially available kit. Blood samples were collected from 257 horses at 19 farms throughout the country and screened for the presence of anti-*B. caballi* antibodies via an indirect immunofluorescent antibody test (IFAT) and for the presence of parasite DNA by nested PCR. The seroprevalence of *B. caballi* was 69.6% and its molecular prevalence was 9.7%. The geographical area, horse’s sex, breed, housing, exposure to ticks, and specifically to *Hyalomma marginatum*, and co-infection with *Theileria equi* were found to be significantly associated with serologic exposure in univariable analysis, while the geographical area and horses’ sex remained significant in the multivariable analysis. The results of this study demonstrate a high level of exposure to *B. caballi* and identify important risk factors for infection. The difference between the serological and molecular prevalence, probably related to parasite clearance, is also highlighted.

## 1. Introduction

*Babesia caballi* is a tick-borne apicomplexan hemoparasite of equines and one of the causative agents of equine piroplasmosis (EP), along with *Theileria equi* [1,2,3]. Equine piroplasmosis is endemic in most parts of the world and poses great concern for the equine industry regarding animal welfare and international horse movement [1,2,3,4]. Clinical disease is mostly attributed to intravascular hemolytic anemia, which may vary in its severity, and usually results in long term carriage of parasites [1,2,3]. Both parasites are usually endemic in the same areas and are vectored by similar species of ticks [5]. *Babesia caballi* is usually less prevalent in most areas, parasitemia in the equine host is usually lower and it tends to cause milder clinical disease than *T. equi.* Moreover, natural parasite clearance without treatment is possible for *B. caballi*, in contrast to *T. equi* [1,2,3,6].

One of the greater challenge EP presents is the identification of carrier horses. This is especially crucial to prevent epizootic spread of the pathogen when horses are transported between endemic and non-endemic regions. Since the parasitemia in carrier horses is low, molecular and serological methods have been developed to increase the sensitivity of the traditional microscopic detection. Currently, the United States Department of Agriculture (USDA)-approved test for screening is a serological competitive enzyme-linked immunosorbent assay (cELISA) based on purified recombinant *B. caballi*, rhoptry associated protein-1 (RAP-1) as an antigen [7]. Nevertheless, although this protein is fairly conserved between isolates, some heterogenicity has been recorded, and the *B. caballi rap-1* cELISA failed to detect infected horses in South Africa and the Middle East, including Israel [8,9,10].

Equine piroplasmosis is endemic in Israel. Several studies have evaluated *T. equi* seroprevalence as 51–66%, its molecular prevalence as 26–56% and identified three prevalent genotypes (18S rRNA genotypes A, C and D) in the area [11,12,13,14,15,16]. However, the seroprevalence of *B. caballi* has never been reported, since the commercial cELISA kit did not yield positive results [10]. The molecular prevalence of *B. caballi* in the area has been estimated as 9.3% by polymerase chain reaction (PCR), more than a decade ago [10]. Restrictions on international movement of horses is based on serology, which is less specific than PCR, but more sensitive. This is attributed to the fact that antibodies remain for prolonged periods of time, while *B. caballi* parasitemia is usually low and temporary since natural clearance can occur [1,3,16]. The aim of this study was to evaluate the serological prevalence of *B. caballi* in horses in Israel by indirect immunofluorescent antibody test (IFAT) using local crude antigen, and to compare it to its molecular prevalence evaluated by nested PCR.

## 2. Results

### 2.1. Study Population

**Figure 1:**
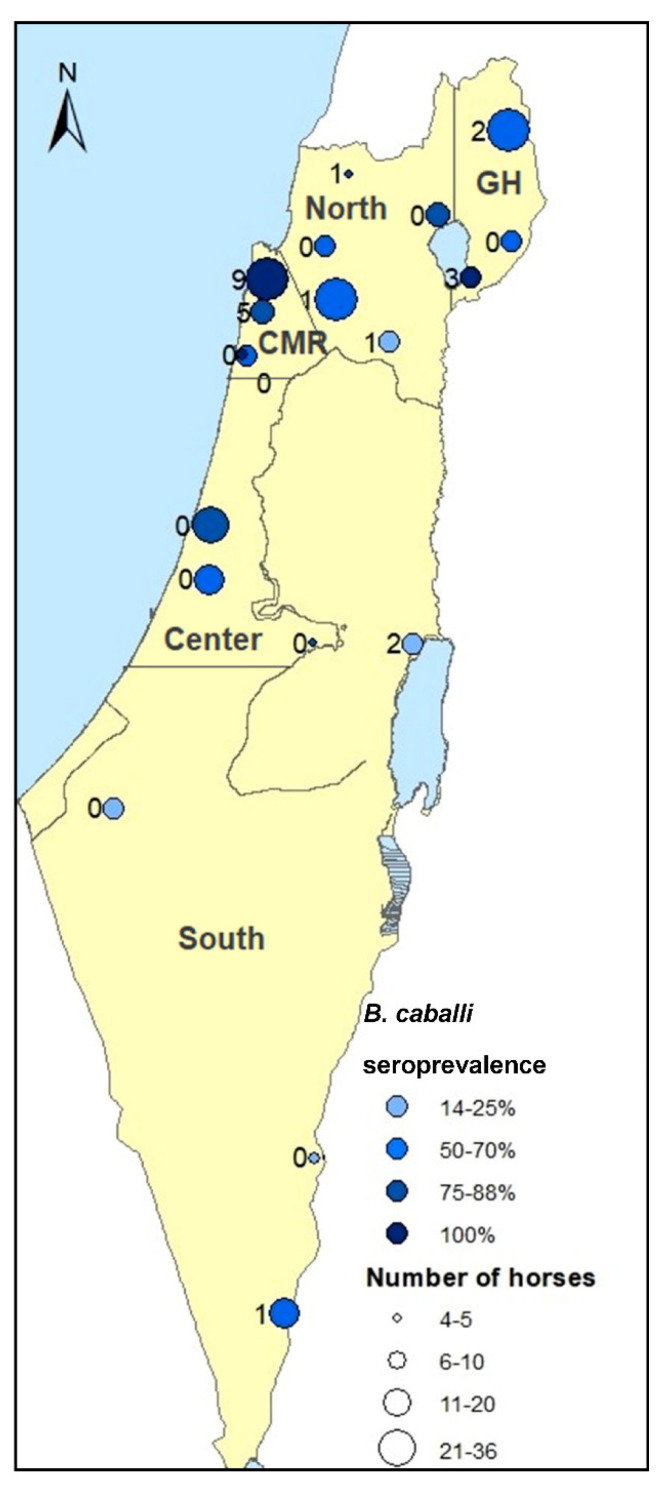
The geographical distribution of the farms included in the study. The number of horses sampled at each farms in represented by the size of the circle. *Babesia caballi* seroprevalence (%), as detected by IFAT, is represented by the intensity of the blue color, while the number of horses infected with *B. caballi*, as tested by nPCR, appears on the left of each point. The division into geographical areas, as used for risk factor analysis appears on the map (GH = Golan heights, CMR = Carmel mountain ridge).

The study population comprised of 257 horses at 19 farms, between 4 and 36 horses at each farm (Figure 1). Of the horses, 123 were mares (47.9%), 128 were geldings (49.8%) and six were stallions (2.3%). Most of the horses were mixed breeds (186/257, 72.4%) and the rest were Quarter Horses (32/257, 12.5%), Arabians (9/257, 3.5%), Paint Horses (9/257, 3.5%), ponies (8/257, 3.1%) and other various breeds (less than 4 horses each). The horses were between nine months and 30 years old (mean = 10.7, standard deviation (SD) = 5.6). Horses were kept only in stalls (46/257, 17.9%), solely or partly in paddocks (91/257, 35.4%) or solely in pasture (120/257, 46.7%). Ticks were found on 98 of the horses during sampling (38.1%), with *Hyalomma marginatum* ticks found on 79 of the horses (30.7%), *Hyalomma excavatum* on 54 horses (21%), *Rhipicephalus turanicus* on 38 horses (14.8%), *Haemaphysalis parva* on five horses (1.9%) and *Rhipicephalus annulatus* on 2 horses (0.8%). Infection with *T. equi* was recorded in 148 (57.6%) of the horses by either PCR or qPCR, and 168 horses (65.4%) were seropositive for *T. equi* by IFAT [17].

### 2.2. Detection of B. caballi Infection by IFAT and PCR

Serologic exposure for *B. caballi* was detected in 179 horses (69.6%, 95% confidence interval (CI): 63.6–75.2), while molecular exposure was detected in 25 horses (9.7%, 95% CI: 6.4–14.0) (Table S1). *Babesia caballi* seroprevalence varied between 14.3% and 100% between farms (Figure 1). Out of the 179 seropositive horses, 19 were also positive by nPCR (10.6%), as well as six of the 78 (7.7%) seronegative horses (Table 1). The agreement between the molecular and the serological detection was weak (K = 0.019, *p* = 0.467).

### 2.3. Risk Factors for Serologic Exposure to B. caballi

Several risk factors were found to be associated with the exposure of horses to *B. caballi*. Horses residing in the area of the Carmel mountain ridge, being of mixed breeds and geldings, infested with ticks and infested with *H. marginatum* ticks had higher risk for being seropositive for *B. caballi*, while horses from the south of Israel and horses kept in paddocks had a lower risk of exposure (univariable analysis; Table 2). Seropositivity was significantly correlated with tick load (ρ = 0.238, *p* < 0.001), but not with horse age (mean age = 10.9 and 10.3 years in seropositive and seronegative horses respectively, *p* = 0.37).

The geographical area, housing management, horses’ breed and sex and tick infestation were significantly associated together (all *p* < 0.001) and therefore, there may have been a confounding effect between them. Nonetheless, only the Carmel mountain ridge and the horses’ sex (gelding) remained significantly associated with *B. caballi* seroprevalence in the multivariable analysis (Table 2).

### 2.4. Risk Factors for Molecular Identification of B. caballi Infection

The risk factors that were positively and significantly associated with the molecular detection of *B. caballi* infection were the geographical area, mixed breed horses, horses infested with ticks and horses infested with *H. marginatum.* Pure bred horses and horses reared in paddocks had a lower risk of infection (Table 3). A significant correlation was found between the horses’ seropositivity and tick load (ρ = 0.165, *p* = 0.008). Age was also significantly associated with the molecular detection of infection (mean age = 8.7 and 11.0 years in positive and negative horses respectively, *p* = 0.038).

The Carmel mountain ridge was the only factor found to be associated with infection in the multivariable analysis (Table 3).

### 2.5. Potential Clinical Consequences of Exposure or Infection

All horses had normal rectal temperature during sample collection (36.5–38.5 °C). Most horses had normal mucus membrane (MM) color, while for 52 horses (20.2%) MM color was noted as pale.

Pale MM was more often noted in seropositive horses (80% versus 66.8%, *p* = 0.052). The distribution of body temperature (*p* = 0.097) and packed cell volume (PCV) (*p* = 0.525) did not differ between seropositive and seronegative horses, while total solids (TS) values were significantly higher in seropositive horses (Mean = 7.2 and 6.9 respectively, *p* = 0.004).

Molecular detection of infection was associated with pale MM (17.3% versus 7.8%, *p* = 0.039). The distribution of body temperature (*p* = 0.54), PCV (*p* = 0.347) and TS (*p* = 0.092) did not differ between positive or negative horses.

## 3. Discussion

Equine piroplasmosis is endemic in the Middle East, and both parasites have been reported in Israel [10], and in neighboring Egypt [9] and Jordan [18,19]. Reports from all three countries stated that the commercial cELISA failed to detect *B. caballi* positive horses, which may have led to underestimation of *B. caballi* prevalence in the area that was generally lower than in Asia or Europe (4.8% versus 25.6% and 12.6% in Asia and Europe respectively) [20]. Further molecular investigation confirmed that *B. caballi* is endemic in the region, and found polymorphism in *rap-1* nucleotide and amino acid sequences, with a considerable difference between African and Middle Eastern isolates, and the American and Caribbean isolates which were used in the development of the cELISA kit [7,8,9,10,19]. This polymorphism may be the reason why the assay based on this protein did not detect a serological response in horses infected by Middle Eastern strains. 

In this study we chose to evaluate *B. caballi* seroprevalence by using IFAT based on a crude antigen of a local isolate, in order to prevent underestimation of the seroprevalence. The results revealed high seroprevalence of *B. caballi* (69.6%), considerably higher than its molecular detection (9.7%). The molecular prevalence in Israel in 2015 was similar to the prevalence in 2007–2008 (9.3%) [10]. While the seroprevalence of *B. caballi* was higher than the seroprevalence of *T. equi* in the same cohort (65.4%), its molecular detection was significantly lower than *T. equi* (57.8%) [17]. These differences are probably attributed to the difference in the epidemiology and transmission dynamics of *T. equi* and *B. caballi.* Since trans–ovarial transmission in vector ticks was documented for *B. caballi*, and since parasite clearance is possible in the equine host, the main reservoir of this parasite is probably the tick vector. On the other hand, *T. equi* carriage is usually life-long, and trans-ovarial transmission was not documented in ticks, making the host (horse) the probable main reservoir [5]. This paradigm fits previous findings, which demonstrated that while *T. equi* prevalence increases with age, the prevalence of *B. caballi*, does not increase with age and is higher in younger animals [21,22]. Our findings concur with this model since age did not correlate with *B. caballi* seroprevalence, but its molecular detection was higher in younger animals, and its seroprevalence was higher than that of *T. equi,* while its molecular detection was lower. These trends were also found in several other serological surveys in endemic areas [23,24,25,26,27,28,29,30,31,32,33,34]. The low molecular detection of *B. caballi* may be attributed to parasite clearance or to low parasitemia, beneath the detection level of the PCR assay. 

Mixed infections of *T. equi* and *B. caballi* have widely been reported worldwide (reviewed in [2]), and were also observed in this study, when the results were compared to a previous study evaluating *T. equi* prevalence in the same cohort [17]. The seroprevalence of *T. equi* in the study population was 65.4%, its molecular prevalence was 57.6%, with an agreement of 0.804 (*p* < 0.001) between methods. Of the 179 *B. caballi* seropositive horses 140 (78.2%) were also seropositive for *T. equi*, as well as 28 of the 78 (35.9%) seronegative horses. Of the 25 *B. caballi* nPCR-positive horses, 20 (80%) were also PCR-positive for *T. equi*, as well as 128 of the 232 negative horses (55.2%). The association between the exposure to both parasites was highly significant (*p* < 0.001). Although co-infection with both parasites is common, little is known regarding possible interactions between them within the equine host and their immune response.

The considerable difference between the presence of anti-*B. caballi* antibodies and the presence of parasite DNA (Table 1) probably reflects the fact that parasitemia may be cleared in some horses, while humoral response with the presence of specific antibodies could remain for long periods, even after clearance. Therefore, it should be taken into account that not all seropositive horses are carriers of parasites. While serological methods cannot discriminate between past exposure and current infection, which may have economic implications on international horse trade or competition, molecular methods may fail to detect infection when the parasitemia is low, and increase the chance of false-negative results.

The geographical area, horses’ breed (mixed bred horses), housing management (pasture) and tick infestation were associated with both *B. caballi* prevalence and seroprevalence, while the horses’ sex (geldings in comparison to mares) was associated only with its seroprevalence and age (younger horses) with its molecular prevalence. A recent meta-analysis of *B. caballi* global distribution and associated risk factors identified higher prevalence in male horses and in younger horses (<5 years) [20]. However, this study did not differentiate between molecular and serological studies when addressing these factors, which may be significant regarding the circulation of this parasite. The main factor that influences exposure of horses to piroplasms is the availability of vector ticks [2,5]. Tick infestation of horses depends on their habitat (geographic and climatic parameters) and housing management (horses reared indoors are usually less exposed). A previous study investigating the population of ticks found on the same cohort of horses throughout the year demonstrated that horses reared in pasture were more prone to be infested with ticks, and had the highest tick loads [35]. Most pasture areas in Israel are located in two geographical areas: the Golan heights and the Carmel mountain ridge (Figure 1), and comparison of the equine tick populations in both areas revealed that the predominant tick species infesting horses in the Golan heights is *H. excavatum*, while in the Carmel mountain ridge it was *H. marginatum* [35]. In the current study, most of the significant risk factors in the univariable analysis associated with each other since mixed-breed and gelded horses are more often kept in pasture (mostly in the Carmel mountain ridge or the Golan heights) and more often infested with ticks. The highest molecular and serological prevalence that were found in horses from the Carmel mountain ridge, where *H. marginatum* is most prevalent, and therefore the geographical area was the main factor to remain significant in the multivariable models. It has been shown that *B. caballi* is transmitted transovarially in *H. marginatum*, but it has not been established for *H. excavatum* [5]. The significantly higher prevalence in the Carmel mountain ridge may suggest better vector competence of *H. marginatum* for transmitting *B. caballi*, which should be further investigated.

Most infected horses in an endemic area are a-symptomatic and may remain carriers for several years [1,2,3]. Investigating possible sub-clinical consequences of infection or exposure revealed that infected horses more often had pale MM than non-infected horses (and this association was close to significant also in seropositive horses, which some may have cleared of infection), although the horses’ PCV was not affected. In addition, seropositive horses had higher TS values, which may be attributed to higher plasma immunoglobulin levels, as demonstrated also for *T. equi* in the same cohort of horses [17].

Limitations of the study included the use of samples collected in 2015, which were selected to enable comparisons to *T. equi* infection and tick infestation, which were available for this cohort [17,35]. Nevertheless, the molecular prevalence in 2015 was similar to the prevalence in 2007–2008 [10]. Further studies are required to determine whether the current prevalence is different. The exact molecular prevalence of *B. caballi,* and any other pathogen for this matter, is limited by the limit of detection of the molecular method used. The limit of detection of the nPCR applied in this study was not determined and, therefore, it is possible that the molecular prevalence is higher. 

## 4. Materials and Methods

### 4.1. Study Design and Sample Collection

A required sample size of 212 horses was calculated based on previously reported *B. caballi* prevalence, using PCR (9.3%) [10] and an estimation of the size of the equine population in Israel (*n* = 35,000).

Nineteen horse farms were sampled to represent the geographical distribution of the equine population in Israel, during May–June 2015. Most of these horses had been previously sampled on three other occasions during 2014–2015, as a part of a seasonal surveillance study [17]. Infection and exposure of these horses to *T. equi* have been evaluated in the same samples (May–June 2015), by PCR and IFAT [17]. Data including individual horse information and farm management were obtained from farm personnel. Whole blood and serum were collected from the jugular vein of each horse into sterile vacuum tubes containing EDTA and without anticoagulant. Serum was obtained after centrifugation (3000 rpm, 10 min), and all blood and serum samples were kept at −20°C until processing. In addition to blood collection, MM color was noted, rectal temperature was measured, and each horse was scanned for the presence of ticks. All ticks were removed and preserved in ethanol and later identified morphologically [35]. 

### 4.2. Serological Screening for B. caballi Exposure

Serological screening for the presence of anti-*B. caballi* antibodies was performed using IFAT. The assay was performed on all sera using a dilution of 1:80, as a cutoff for screening. The antigen slides were prepared in-house from *B. caballi*-infected erythrocytes from culture. Blood smears with parasitemia of 5% or higher were prepared on glass slides and stored at −80 °C until use. Test protocol was performed as previously described for *T. equi* [13,36].

### 4.3. Identification of B. caballi Infection Using Nested PCR 

DNA was extracted from whole blood of each sample and infection with *B. caballi* was identified by nested PCR directed at the *B. caballi rap-1* gene, using the external primers BC-RAPF and BC-RAP1R (amplifying~1800 bp), and the internal primers BC-RAP2F and BC-RAP2R (amplifying~400 bp), as previously described [8,16]. 

### 4.4. Statistical Analysis

The agreement between IFAT and nPCR results for identification of *B. caballi* was assessed using Cohen’s Kappa. 

Risk factors for serologic exposure to *B. caballi* were evaluated using χ^2^ or Fisher’s exact test, as appropriate, for categorical parameters, and using t-test or Mann–Whitney U-test, as appropriate, for scale parameters. Odds ratios were calculated using univariable logistic regression models for each parameter. All factors that were found significant in the univariable analysis were included in a multivariable generalized estimating equation (GEE), with a logit link function, the horse was defined as the subject and the farm was defined as a random factor.

All statistical analyses were performed using SPSS 25.0^®^ and WinPepi 11.65^®^ statistical software. Statistical significance was set at *p* < 0.05. 

## 5. Conclusions

The use of local crude antigen proved efficient for the evaluation of the seroprevalence of *B. caballi* in Israel, and may be beneficial for pre-exportation screening to non-endemic areas. While the molecular prevalence was similar to previous reports, the serological prevalence was considerably higher, suggesting that most horses in the studied area are exposed to this parasite. Risk factor analysis identified one geographical area with the highest rates of infection and exposure, that may be linked to higher prevalence of *H. marginatum* ticks.

## Figures and Tables

**Table 1:** Detection of *B. caballi* exposure or infection by IFAT and nPCR in 257 horses from 19 farms in Israel.

	nPCR-Negative (%)	nPCR-Positive (%)	Total
IFAT-Negative	72 (92.3)	6 (7.7)	78
IFAT-Positive	160 (89.4)	19 (10.6)	179
Total	232	25	257

**Table 2:** Factors associated with serologic exposure to *B. caballi* in horses in Israel, as detected by an immunofluorescent antibody test (IFAT). Univariable analysis was performed by Chi square test or Fisher’s exact, as appropriate. Multivariable analysis was performed using generalized estimating equation (GEE) with the farm defined as a random factor and included all factors identified as significant in the univariable analysis. Odds ratios (OR) were calculated from the GEE model. Statistically significant associations appear in bold.

	N	IFAT-Positive (%)	*p* (χ^2^)	*p* (GEE)	OR (95% CI)
North	69	40 (58)	**<0.001**	0.138	3.45 (0.67–17.77)
Carmel	64	59 (92.2)		**<0.001**	10.01 (2.98–33.59)
Center	47	35 (74.5)		0.062	5.45 (0.92–32.30)
South	35	14 (40)		0.227	3.11 (0.49–19.57)
Golan heights	42	31 (73.8)			
Gelding	128	103 (80.5)	**<0.001**	**0.037**	2.01 (1.04–3.89)
Stallion	6	4 (66.7)		0.325	2.61 (0.39–17.54)
Mare	123	72 (58.5)		ref	
Mixed Breed	186	142 (76.3)	**<0.001**	0.234	1.58 (0.74–3.33)
Pure breed	70	36 (51.4)			
Stall	46	36 (78.3)	**<0.001**	0.841	0.85 (0.16–4.33)
Paddock	91	41 (45.1)		0.064	0.27 (0.07–1.08)
Pasture	120	102 (85)		ref	
Ticks	98	84 (85.7)	**<0.001**	0.127	6.98 (0.58–84.34)
No ticks	159	95 (59.7)		ref	
*Haemaphysalis parva*	5	4 (80)	1		
Not present	252	175 (69.4)			
*Hyalomma excavatum*	54	41 (75.9)	0.259		
Not present	203	138 (68)			
*Hyalomma marginatum*	79	67 (84.8)	**<0.001**	0.191	2.63 (0.62–11.20)
Not present	178	112 (62.9)			
*Rhipicephalus annulatus*	2	2 (100)	1		
Not present	255	177 (69.4)			
*Rhipicephalus turanicus*	38	28 (73.7)	0.558		
Not present	219	151 (68.9)			

**Table 3:** Factors associated with molecular exposure to *B. caballi* in horses in Israel, as detected by nested polymerase chain reaction (nPCR). Univariable analysis was performed by Chi square test or Fisher’s exact, as appropriate. Multivariable analysis was performed using a generalized estimating equation (GEE) with the farm defined as a random factor and included all factors identified as significant in the univariable analysis. Odds ratios (OR) were calculated from the GEE model. Statistically significant associations appear in bold.

	N	nPCR-Positive (%)	*p* (χ^2^)	*p* (GEE)	OR (95% CI)
North	69	3 (4.3)	0.001	0.798	0.81 (0.16–4.01)
Carmel	64	14 (21.9)		**0.025**	3.82 (1.19–12.32)
Center	47	0		na	na
South	35	3 (8.6)		0.686	1.62 (0.16–16.89)
Golan heights	42	5 (11.9)		ref	
Gelding	128	15 (11.7)	0.284		
Stallion	6	1 (16.7)			
Mare	123	9 (7.3)			
Mixed Breed	186	24 (12.9)	**0.006**	0.083	9.86 (0.74–130.92)
Pure breed	70	1 (1.4)		ref	
Stall	46	0	**0.001**	na	na
Paddock	91	5 (5.5)		0.916	1.11 (0.16–7.79)
Pasture	120	20 (16.7)		ref	
Ticks	98	16 (16.3)	**0.005**	0.667	1.34 (0.31–6.36)
No ticks	159	9 (5.7)		ref	
*Haemaphysalis parva*	5	0	1		
Not present	252	25 (9.9)			
*Hyalomma excavatum*	54	7 (13)	0.367		
Not present	203	18 (8.9)			
*Hyalomma marginatum*	79	13 (16.5)	**0.015**	0.528	0.624 (0.14–2.69)
Not present	178	12 (6.7)		ref	
*Rhipicephalus annulatus*	2	0	1		
Not present	255	25 (9.8)			
*Rhipicephalus turanicus*	38	5 (13.2)	0.388		
Not present	219	20 (9.1)			
Age				0.051	

## Data Availability

Data are contained within the article or Supplementary Material.

## Supplementary Materials

The following are available online at https://www.mdpi.com/article/10.3390/pathogens10040445/s1, Table S1: Data of the horses included in this study including their characteristics, management, tick infestation, clinical parameters and the molecular and serological results of *B. caballi* and *T. equi* infection.
