# Supplementary material for: Serological and Molecular Prevalence of Babesia caballi in Apparently Healthy Horses in Israel
Source: Pathogens. 2021 Apr 8;10(4):445. doi: 10.3390/pathogens10040445 (PMC8068206; doi:10.3390/pathogens10040445)
Supplement: Supplementary file 1 [file pathogens-10-00445-s001.pdf]

Farm	Farm Location	Area	Horse ID	Age	Breed	Gender	Housing	Date	Temp	MM	Ticks 01	Total ticks	HP	HE	HM	RA	RT	PCV	TS	TE-PCR	TE-IFAT	BC-IFAT	BC-nPCR
1	Kerem Maharal	Carmel	A1	17	Mixed	Gelding	Pasture	3.6.15	37.3	Pale	0	0	0	0	0	0	0	35	7.2	1	1	1	0
1	Kerem Maharal	Carmel	A2	20	Mixed	Gelding	Pasture	3.6.15	37.7	Pale	1	2	0	0	2	0	0	32	6.8	1	1	1	0
1	Kerem Maharal	Carmel	A3	18	Mixed	Gelding	Pasture	3.6.15	37.6	Pale	0	0	0	0	0	0	0	30	7.6	1	1	1	0
1	Kerem Maharal	Carmel	A4	18	Mixed	Gelding	Pasture	3.6.15	37.4	Pink	0	0	0	0	0	0	0	35	7	1	1	1	0
1	Kerem Maharal	Carmel	A5	28	Mixed	Gelding	Pasture	3.6.15	37.3	Pale	1	6	0	0	5	1	0	31	7	1	1	1	0
1	Kerem Maharal	Carmel	A6	7	Mixed	Gelding	Pasture	3.6.15	37.3	Pink	0	0	0	0	0	0	0	32	7.2	1	1	1	0
1	Kerem Maharal	Carmel	A7	7	Mixed	Gelding	Pasture	3.6.15	37.6	Pink	0	0	0	0	0	0	0	33	7	1	1	1	1
1	Kerem Maharal	Carmel	A9	5	Mixed	Mare	Stall	3.6.15	37.2	Pink	0	0	0	0	0	0	0	32	7.2	1	1	1	0
1	Kerem Maharal	Carmel	A10	18	Mixed	Gelding	Pasture	3.6.15	37.4	Pink	1	1	0	0	1	0	0	33	6.4	1	1	1	0
1	Kerem Maharal	Carmel	A11	4	Mixed	Gelding	Stall	3.6.15	37.1	Pink	0	0	0	0	0	0	0	28	7	1	1	1	1
1	Kerem Maharal	Carmel	A12	2	Mixed	Gelding	Paddock	3.6.15	37.6	Pink	0	0	0	0	0	0	0	30	6.8	1	1	1	0
1	Kerem Maharal	Carmel	A13	2	Mixed	Gelding	Paddock	3.6.15	37.6	Pink	1	1	0	0	1	0	0	40	8.4	1	1	1	0
1	Kerem Maharal	Carmel	A14	3	Mixed	Gelding	Paddock	3.6.15	37.3	Pink	0	0	0	0	0	0	0	32	6.6	1	1	1	1
1	Kerem Maharal	Carmel	A15	10	Mixed	Gelding	Paddock	3.6.15	36.7	Pink	0	0	0	0	0	0	0	38	7.4	0	0	0	1
1	Kerem Maharal	Carmel	A16	10	Mixed	Gelding	Paddock	3.6.15	37.1	Pink	0	0	0	0	0	0	0	38	6.8	0	0	0	0
1	Kerem Maharal	Carmel	A17	15	Mixed	Gelding	Paddock	3.6.15	37.6	Pale	1	2	0	0	2	0	0	39	7.4	1	1	1	1
2	Binyamina	Carmel	B2	18	Mixed	Mare	Pasture	3.6.15	37.6	Pale	0	0	0	0	0	0	0	32	6.6	1	1	0	0
2	Binyamina	Carmel	B3	20	Mixed	Mare	Pasture	3.6.15	36.1	Pale	0	0	0	0	0	0	0	39	6.6	1	1	1	0
2	Binyamina	Carmel	B4	6	Foxtrot	Mare	Paddock	3.6.15	37.3	Pink	1	1	0	1	0	0	0	32	6.2	0	0	1	0
2	Binyamina	Carmel	B5	10	Paint	Mare	Paddock	3.6.15	37.6	Pink	0	0	0	0	0	0	0	38	6.8	0	0	1	0
2	Binyamina	Carmel	B6	7	Quarter	Gelding	Paddock	3.6.15	36.8	Pink	0	0	0	0	0	0	0	33	6.4	0	0	1	0
2	Binyamina	Carmel	B8	18	Paint	Gelding	Stall	3.6.15	36.8	Pink	0	0	0	0	0	0	0	29	6.2	0	0	0	0
2	Binyamina	Carmel	B9	4	Quarter	Gelding	Stall	3.6.15	37.2	Pink	0	0	0	0	0	0	0	39	6.2	0	0	0	0
2	Binyamina	Carmel	B10			Mare		3.6.15	37.1	Pink	0	0	0	0	0	0	0	38	6.8	1	1	1	0
3	Beit Hananaya	Carmel	C1	10	Mixed	Gelding	Stall	3.6.15	37.2	Pink	0	0	0	0	0	0	0	28	7.2	0	0	1	0
3	Beit Hananaya	Carmel	C2	7	Mixed	Mare	Stall	3.6.15	38.3	Pink	0	0	0	0	0	0	0	36	7	1	1	1	0
3	Beit Hananaya	Carmel	C3	17	Warmblood	Gelding	Stall	3.6.15	37.6	Pink	0	0	0	0	0	0	0	35	7.2	0	0	1	0
3	Beit Hananaya	Carmel	C5	10	Warmblood	Gelding	Stall	3.6.15	37	Pink	0	0	0	0	0	0	0	37	7.2	0	1	1	0
4	Beit Oren	Carmel	D1		Mixed	Gelding	Pasture	3.6.15	36.5	Pale	1	12	0	0	12	0	0	34	7.6	1	1	1	0
4	Beit Oren	Carmel	D2		Mixed	Gelding	Pasture	3.6.15	37.1	Pale	1	12	0	0	12	0	0	33	7.2	1	1	1	0
4	Beit Oren	Carmel	D3		Mixed	Gelding	Pasture	3.6.15	36.9	Pink	1	1	0	0	1	0	0	32	7	1	1	1	0
4	Beit Oren	Carmel	D4	2	Mixed	Gelding	Pasture	3.6.15	37.4	Pale	1	13	0	1	12	0	0	27	7.2	1	1	1	0
4	Beit Oren	Carmel	D7	20	Mixed	Gelding	Pasture	3.6.15	36.4	Pale	1	7	0	0	7	0	0	31	6.6	1	1	1	1
4	Beit Oren	Carmel	D11	5	Mixed	Mare	Pasture	3.6.15	36.1	Pink	1	11	0	0	11	0	0	29	7.2	1	1	1	0
4	Beit Oren	Carmel	D13	5	Mixed	Gelding	Pasture	3.6.15	37	Pink	1	10	0	0	10	0	0	32	7.4	1	1	1	0
4	Beit Oren	Carmel	D14	8	Mixed	Gelding	Pasture	3.6.15	37.2	Pale	1	10	0	0	10	0	0	32	7.4	1	1	1	0
4	Beit Oren	Carmel	D15	10	Mixed	Mare	Pasture	3.6.15	37.1	Pink	1	10	0	0	10	0	0	35	7.4	1	1	1	1
4	Beit Oren	Carmel	D18	12	Mixed	Gelding	Pasture	3.6.15	36	Pale	1	13	0	0	13	0	0	30	7	1	1	1	0
4	Beit Oren	Carmel	D19	9	Mixed	Gelding	Pasture	3.6.15	36.8	Pink	1	5	0	0	5	0	0	32	6.8	1	1	1	0

4	Beit Oren	Carmel	D20	10	Mixed	Gelding	Pasture	3.6.15	36	Pink	1	6	0	0	6	0	0	32	7.8	1	1	1	0
4	Beit Oren	Carmel	D21	12	Mixed	Mare	Pasture	3.6.15	37.2	Pink	1	9	0	0	9	0	0	33	6.8	1	1	1	0
4	Beit Oren	Carmel	D24	5	Mixed	Gelding	Pasture	3.6.15	37.2	Pale	1	10	0	0	10	0	0	35	7	1	1	1	0
4	Beit Oren	Carmel	D25	11	Mixed	Gelding	Pasture	3.6.15	36.8	Pink	1	8	0	0	8	0	0	34	7	1	1	1	0
4	Beit Oren	Carmel	D26	8	Mixed	Mare	Pasture	3.6.15	37	Pale	1	6	0	0	6	0	0	35	7.4	1	1	1	0
4	Beit Oren	Carmel	D27	15	Mixed	Mare	Pasture	3.6.15	36.2	Pink	1	4	0	0	4	0	0	33	7	1	1	1	0
4	Beit Oren	Carmel	D28	10	Mixed	Mare	Pasture	3.6.15	37.1	Pink	1	12	0	0	12	0	0	33	7.6	1	1	1	0
4	Beit Oren	Carmel	D29	15	Mixed	Mare	Pasture	3.6.15	36.1	Pink	1	13	0	1	12	0	0	39	7.5	1	1	1	0
4	Beit Oren	Carmel	D30	10	Mixed	Mare	Pasture	3.6.15	37	Pale	1	6	0	0	6	0	0	35	7.4	1	1	1	1
4	Beit Oren	Carmel	D31	8	Mixed	Gelding	Pasture	3.6.15	37.1	Pink	1	23	0	0	23	0	0	33	7.8	1	1	1	0
4	Beit Oren	Carmel	D32	8	Mixed	Gelding	Pasture	3.6.15	36.3	Pink	1	7	0	1	6	0	0	36	7.8	1	1	1	1
4	Beit Oren	Carmel	D33	6	Mixed	Gelding	Pasture	3.6.15	37	Pink	1	4	0	0	4	0	0	35	7	1	1	1	1
4	Beit Oren	Carmel	D35	8	Mixed	Gelding	Pasture	3.6.15	37.2	Pale	1	12	0	0	12	0	0	30	7.4	1	1	1	1
4	Beit Oren	Carmel	D36	18	Mixed	Mare	Pasture	3.6.15	37.5	Pink	1	9	0	0	8	0	0	30	7.6	1	1	1	0
4	Beit Oren	Carmel	D37	25	Mixed	Gelding	Pasture	3.6.15	37.2	Pink	1	2	0	0	2	0	0	33	7.4	1	1	1	0
4	Beit Oren	Carmel	D38	8	Mixed	Gelding	Pasture	3.6.15	36.4	Pale	1	4	0	0	4	0	0	34	7.4	1	1	1	1
4	Beit Oren	Carmel	D39	9	Mixed	Mare	Pasture	3.6.15	36.6	Pink	1	21	0	0	21	0	0	34	7.2	1	1	1	0
4	Beit Oren	Carmel	D40	9	Mixed	Mare	Pasture	3.6.15	37.3	Pale	1	3	0	0	3	0	0	35	7.2	1	1	1	0
4	Beit Oren	Carmel	D41	23	Mixed	Mare	Pasture	3.6.15	37.1	Pale	1	2	0	0	2	0	0	33	7.2	1	1	1	0
4	Beit Oren	Carmel	D42	14	Mixed	Mare	Pasture	3.6.15	36	Pink	1	2	0	0	2	0	0	33	7.2	1	1	1	0
4	Beit Oren	Carmel	D43	3	Mixed	Gelding	Pasture	3.6.15	37.2	Pink	1	5	0	0	5	0	0	27	7.6	1	1	1	0
4	Beit Oren	Carmel	D44	13	Mixed	Mare	Pasture	3.6.15	37.2	Pink	1	4	0	0	4	0	0	32	7.4	1	1	1	0
4	Beit Oren	Carmel	D45	12	Mixed	Mare	Pasture	3.6.15	37	Pink	1	2	0	0	2	0	0	33	7.2	0	1	1	0
4	Beit Oren	Carmel	D46	2	Mixed	Gelding	Pasture	3.6.15	37	Pale	1	17	0	1	16	0	0	32	6.6	1	1	1	1
4	Beit Oren	Carmel	D47	13	Mixed	Mare	Pasture	3.6.15	37.8	Pale	1	10	0	0	10	0	0	31	7.4	1	1	1	1
5	Ramat Gan	Center	E1	10	Mixed	Gelding	Stall	13.5.15	36.7	Pink	0	0	0	0	0	0	0	40	6.8	1	1	1	0
5	Ramat Gan	Center	E2	15	Pony	Mare	Stall	13.5.15	36.8	Pink	0	0	0	0	0	0	0	47	7.2	1	1	1	0
5	Ramat Gan	Center	E3	12	Mixed	Mare	Stall	13.5.15	37.1	Pink	0	0	0	0	0	0	0	38	6.8	0	0	1	0
5	Ramat Gan	Center	E5	13	Mixed	Mare	Stall	13.5.15	36.8	Pink	0	0	0	0	0	0	0	45	7	0	0	0	0
5	Ramat Gan	Center	E6	9	Mixed	Gelding	Stall	13.5.15	37	Pale	0	0	0	0	0	0	0	32	6	0	0	1	0
5	Ramat Gan	Center	E8	12	Mixed	Mare	Stall	13.5.15	37.6	Pink	0	0	0	0	0	0	0	34	7	0	0	1	0
5	Ramat Gan	Center	E9	17	Mixed	Gelding	Stall	13.5.15	36.6	Pink	0	0	0	0	0	0	0	30	7.2	0	0	1	0
5	Ramat Gan	Center	E10	12	Mixed	Gelding	Stall	13.5.15	37.2	Pale	0	0	0	0	0	0	0	34	6.4	0	0	1	0
5	Ramat Gan	Center	E13	20	Mixed	Gelding	Stall	13.5.15	37.1	Pink	0	0	0	0	0	0	0	38	6	1	1	1	0
5	Ramat Gan	Center	E16	13	Mixed	Mare	Stall	13.5.15	37.3	Pink	0	0	0	0	0	0	0	38	6.6	1	1	1	0
5	Ramat Gan	Center	E17	12	Mixed	Gelding	Stall	13.5.15	37.1	Pink	0	0	0	0	0	0	0	30	6.4	0	1	1	0
5	Ramat Gan	Center	E18	20	Mixed	Gelding	Stall	13.5.15	36.4	Pink	0	0	0	0	0	0	0	37	7	0	1	0	0
5	Ramat Gan	Center	E19	15	Mixed	Mare	Stall	13.5.15	37.4	Pink	0	0	0	0	0	0	0	34	7.4	1	1	1	0
5	Ramat Gan	Center	E20	5	Mixed	Gelding	Stall	13.5.15	37.7	Pink	0	0	0	0	0	0	0	30	6	0	0	1	0
5	Ramat Gan	Center	E21	7	Pony	Mare	Stall	13.5.15	36.3	Pink	0	0	0	0	0	0	0	39	6.6	0	1	0	0
5	Ramat Gan	Center	E23	11	Pony	Mare	Stall	13.5.15	37.1	Pink	0	0	0	0	0	0	0	35	7.4	0	1	1	0

5	Ramat Gan	Center	E27	17	Mixed	Gelding	Stall	13.5.15	37.6	Pink	0	0	0	0	0	0	0	33	6.4	1	0	0	0
5	Ramat Gan	Center	E28	19	Mixed	Gelding	Stall	13.5.15	36.1	Pink	0	0	0	0	0	0	0	35	6	0	0	1	0
5	Ramat Gan	Center	E29	8	Tinker	Gelding	Stall	13.5.15	37.9	Pink	0	0	0	0	0	0	0	32	6.4	0	0	1	0
5	Ramat Gan	Center	E30	15	Mixed	Mare	Stall	13.5.15	37.4	Pink	0	0	0	0	0	0	0	31	5.8	1	1	1	0
5	Ramat Gan	Center	E32	20	Mixed	Mare	Stall	13.5.15	37.4	Pink	0	0	0	0	0	0	0	31	6.6	0	0	1	0
5	Ramat Gan	Center	E33	6	Tinker	Gelding	Stall	13.5.15	37.2	Pink	0	0	0	0	0	0	0	28	6.4	0	0	1	0
5	Ramat Gan	Center	E34	11	Mixed	Gelding	Stall	13.5.15	37.1	Pink	0	0	0	0	0	0	0	35	6.8	0	0	1	0
5	Ramat Gan	Center	E35	17	Mixed	Gelding	Stall	13.5.15	36.4	Pink	0	0	0	0	0	0	0	37	6.4	0	0	1	0
5	Ramat Gan	Center	E36	12	Mixed	Gelding	Stall	13.5.15	37.2	Pink	0	0	0	0	0	0	0	38	7.4	0	0	1	0
5	Ramat Gan	Center	E37	22	Pony	Stallion	Stall	13.5.15	37.1	Pale	0	0	0	0	0	0	0	35	6.6	1	1	1	0
6	Nezer Sireni	Center	F2	8	Mixed	Gelding	Paddock	17.5.15	36.8	Pale	0	0	0	0	0	0	0	30	6.2	1	0	0	0
6	Nezer Sireni	Center	F3	9	Mixed	Mare	Paddock	17.5.15	37.4	Pale	0	0	0	0	0	0	0	30	6.8	0	0	0	0
6	Nezer Sireni	Center	F5	10	Pony	Mare	Paddock	17.5.15	37.6	Pink	0	0	0	0	0	0	0	32	6.8	0	0	0	0
6	Nezer Sireni	Center	F6	18	Mixed	Gelding	Paddock	17.5.15	37.5	Pink	0	0	0	0	0	0	0	29	6.8	0	0	1	0
6	Nezer Sireni	Center	F7	7	Mixed	Gelding	Paddock	17.5.15	37.2	Pale	0	0	0	0	0	0	0	29	6.2	0	0	0	0
6	Nezer Sireni	Center	F8	10	Mixed	Gelding	Paddock	17.5.15	37.2	Pale	0	0	0	0	0	0	0	34	6.8	1	1	1	0
6	Nezer Sireni	Center	F9	15	Pony	Mare	Paddock	17.5.15	37.1	Pale	0	0	0	0	0	0	0	32	6.2	0	0	0	0
6	Nezer Sireni	Center	F10	12	Mixed	Mare	Paddock	17.5.15	37.8	Pink	0	0	0	0	0	0	0	29	7.4	0	0	1	0
6	Nezer Sireni	Center	F11	12	Mixed	Gelding	Paddock	17.5.15	37	Pink	0	0	0	0	0	0	0	35	7.2	0	0	1	0
6	Nezer Sireni	Center	F12	9	Mixed	Mare	Paddock	17.5.15	37.2	Pink	0	0	0	0	0	0	0	37	6.8	1	1	1	0
6	Nezer Sireni	Center	F13	17	Mixed	Gelding	Paddock	17.5.15	36.8	Pale	0	0	0	0	0	0	0	35	6.8	1	1	1	0
6	Nezer Sireni	Center	F14	12	Mixed	Mare	Paddock	17.5.15	37.4	Pink	0	0	0	0	0	0	0	34	6.8	1	1	1	0
6	Nezer Sireni	Center	F16	12	Mixed	Gelding	Stall	17.5.15	38.2	Pink	0	0	0	0	0	0	0	35	7	1	1	0	0
7	Ora	Center	G1	17	Mixed	Mare	Stall	15.6.15	36.9	Pale	0	0	0	0	0	0	0	40	7.6	0	0	0	0
7	Ora	Center	G2	13	Paint	Gelding	Stall	15.6.15	37	Pink	0	0	0	0	0	0	0	26	8.6	0	0	1	0
7	Ora	Center	G4	12	Mixed	Gelding	Stall	15.6.15	37	Pink	0	0	0	0	0	0	0	39	8	0	1	1	0
7	Ora	Center	G5	9	Mixed	Gelding	Stall	15.6.15	37.2	Pink	0	0	0	0	0	0	0	36	6.6	0	0	0	0
7	Ora	Center	G6	16	Quarter	Gelding	Stall	15.6.15	37.1	Pink	0	0	0	0	0	0	0	40	7.8	1	1	1	0
7	Ora	Center	G8	12	Mixed	Gelding	Stall	15.6.15	37.1	Pink	0	0	0	0	0	0	0	37	7.4	0	1	1	0
7	Ora	Center	G12	18	Mixed	Gelding	Stall	15.6.15	37.2	Pink	0	0	0	0	0	0	0	35	8.2	0	1	1	0
7	Ora	Center	G13	11	Mixed	Gelding	Stall	15.6.15	36.8	Pink	0	0	0	0	0	0	0	37	7	0	1	1	0
8	Kalia	South	H1	13	Shire	Mare	Paddock	27.5.15	37.6	Pink	0	0	0	0	0	0	0	29	7	0	1	1	0
8	Kalia	South	H2	13	Quarter	Mare	Paddock	27.5.15	37.6	Pink	0	0	0	0	0	0	0	31	6.2	0	1	0	0
8	Kalia	South	H4	8	Quarter	Mare	Paddock	27.5.15	37.4	Pink	0	0	0	0	0	0	0	38	6.2	1	1	0	0
8	Kalia	South	H5	8	Quarter	Mare	Paddock	27.5.15	37.5	Pink	0	0	0	0	0	0	0	35	6.6	0	0	1	0
8	Kalia	South	H6	7	Mixed	Gelding	Paddock	27.5.15	37.6	Pink	0	0	0	0	0	0	0	32	6.6	0	0	0	1
8	Kalia	South	H7	13	Paint	Gelding	Paddock	27.5.15	37.5	Pink	0	0	0	0	0	0	0	33	6.8	0	0	0	0
8	Kalia	South	H11	0.75	Quarter	Mare	Paddock	27.5.15		Pink	0	0	0	0	0	0	0	37	6.4	0	0	0	0
8	Kalia	South	H12	1.5	Arabian	Mare	Stall	27.5.15	37.8	Pink	0	0	0	0	0	0	0	33	6.2	0	0	0	0
8	Kalia	South	H13	12	Quarter	Mare	Paddock	27.5.15	38	Pink	0	0	0	0	0	0	0	32	8	0	0	0	1
9	Ifat	North	I1	5	Quarter	Mare	Stall	15.6.15	37.9	Pink	0	0	0	0	0	0	0	37	6.4	0	0	0	0

9	Ifat	North	I2	7	Quarter	Gelding	Stall	15.6.15	37.4	Pale	0	0	0	0	0	0	0	32	6.9	1	1	1	0
9	Ifat	North	I4	5	Pony	Gelding	Stall	15.6.15	36.7	Pink	0	0	0	0	0	0	0	36	6.8	0	0	0	0
9	Ifat	North	I5	9	Appaloosa	Mare	Stall	15.6.15	37.4	Pink	0	0	0	0	0	0	0	37	7	1	1	1	0
9	Ifat	North	I6	5	Appaloosa	Mare	Stall	15.6.15	37.9	Pink	0	0	0	0	0	0	0	32	7	0	0	0	0
9	Ifat	North	I7	14	Mixed	Mare	Stall	15.6.15	37.4	Pink	0	0	0	0	0	0	0	35	7.4	0	0	0	0
9	Ifat	North	I8	5	Mixed	Gelding	Stall	15.6.15	37.2	Pink	0	0	0	0	0	0	0	32	7.2	1	1	1	0
9	Ifat	North	I9	14	Mixed	Gelding	Stall	15.6.15	37.5	Pale	0	0	0	0	0	0	0	36	7	1	1	1	0
9	Ifat	North	I10	15	Mixed	Mare	Stall	15.6.15	37.1	Pink	0	0	0	0	0	0	0	36	7.4	1	1	1	0
9	Ifat	North	I11	15	Mixed	Mare	Stall	15.6.15	37.1	Pale	0	0	0	0	0	0	0	35	6	0	0	0	0
9	Ifat	North	I12	7	Quarter	Gelding	Stall	15.6.15	37.2	Pink	0	0	0	0	0	0	0	40	6.4	1	1	1	0
9	Ifat	North	I15	7	Quarter	Gelding	Stall	15.6.15	37	Pink	0	0	0	0	0	0	0	34	6.2	1	1	0	0
9	Ifat	North	I16	5	Quarter	Mare	Paddock	15.6.15	37	Pink	0	0	0	0	0	0	0	38	7.4	0	0	0	0
9	Ifat	North	I17	6	Quarter	Gelding	Paddock	15.6.15	37.3	Pink	0	0	0	0	0	0	0	36	7.4	0	0	0	0
9	Ifat	North	I18	7	Mixed	Gelding	Paddock	15.6.15	37.1	Pink	0	0	0	0	0	0	0	38	6.8	1	1	1	0
9	Ifat	North	I19	12	Paint	Gelding	Paddock	15.6.15	37	Pink	0	0	0	0	0	0	0	35	6.4	0	0	1	0
9	Ifat	North	I20	10	Appaloosa	Mare	Paddock	15.6.15	37.4	Pink	0	0	0	0	0	0	0	31	7.2	0	1	1	0
9	Ifat	North	I21	2	Quarter	Mare	Paddock	15.6.15	37.5	Pink	0	0	0	0	0	0	0	42	6.2	0	0	0	0
9	Ifat	North	I22	2	Quarter	Mare	Paddock	15.6.15	37.9	Pink	0	0	0	0	0	0	0	40	6.8	0	0	0	0
9	Ifat	North	I23	13	Quarter	Mare	Paddock	15.6.15	36.8	Pink	0	0	0	0	0	0	0	38	7.4	0	0	0	0
9	Ifat	North	I24	17	Appaloosa	Gelding	Paddock	15.6.15	37	Pink	0	0	0	0	0	0	0	35	7.2	1	1	1	0
9	Ifat	North	I25	1	Quarter	Mare	Paddock	15.6.15	37.8	Pink	0	0	0	0	0	0	0	32	6.6	0	0	1	0
9	Ifat	North	I26	22	Paint	Stallion	Paddock	15.6.15	37.1	Pink	0	0	0	0	0	0	0	38	7.6	0	0	1	0
9	Ifat	North	I27	15	Paint	Mare	Paddock	15.6.15	36.8	Pink	0	0	0	0	0	0	0	38	6.8	0	0	1	0
9	Ifat	North	I28	10	Quarter	Mare	Paddock	15.6.15	37.4	Pink	0	0	0	0	0	0	0	41	7.4	0	1	0	0
9	Ifat	North	I30	15	Quarter	Mare	Paddock	15.6.15	37.2	Pink	0	0	0	0	0	0	0	33	7.4	1	1	0	0
9	Ifat	North	I31	2	Mixed	Stallion	Paddock	15.6.15	37.7	Pink	0	0	0	0	0	0	0	25	7.6	1	1	1	1
9	Ifat	North	I32	3	Paint	Stallion	Paddock	15.6.15	37.3	Pink	0	0	0	0	0	0	0	37	6.4	0	0	0	0
11	Maona	North	K1	21	Mixed	Gelding	Pasture	28.5.15	36.8	Pink	1	4	0	0	4	0	0	35	7.6	1	1	1	0
11	Maona	North	K2	14	Mixed	Mare	Pasture	28.5.15	37.2	Pale	1	9	0	1	8	0	0	39	7	1	1	1	0
11	Maona	North	K3	13	Mixed	Mare	Pasture	28.5.15	37.4	Pink	1	4	0	0	4	0	0	34	7	1	1	1	0
11	Maona	North	K4	10	Mixed	Gelding	Pasture	28.5.15	37.2	Pink	1	1	0	0	1	0	0	38	7.6	1	1	0	0
11	Maona	North	K6	12	Quarter	Gelding	Pasture	28.5.15	37.2	Pink	1	1	0	0	1	0	0	39	7	1	1	1	0
11	Maona	North	K10	4	Mixed	Gelding	Pasture	28.5.15		Pink	1	2	0	0	2	0	0	26	8.2	1	1	1	0
11	Maona	North	K11	10	Mixed	Gelding	Pasture	28.5.15	36.9	Pink	0	0	0	0	0	0	0	35	8.2	1	1	1	0
11	Maona	North	K13	5	Mixed	Mare	Pasture	28.5.15	37.2	Pink	1	8	0	0	8	0	0	36	8.6	1	1	1	1
11	Maona	North	K14	10	Paint	Gelding	Pasture	28.5.15	37.4	Pink	0	0	0	0	0	0	0	34	8	0	0	0	0
12	NNov	Golan H	L2	14	Mixed	Mare	Pasture	6.5.15	37.7	Pale	1	8	0	1	4	0	3	35	8.6	1	1	1	0
12	NNov	Golan H	L3	9	Mixed	Gelding	Pasture	6.5.15	37.2	Pink	1	13	0	10	0	0	3	30	8.2	0	1	1	0
12	NNov	Golan H	L4	9	Mixed	Gelding	Pasture	6.5.15	37	Pink	1	27	0	11	7	0	9	33	7.6	1	1	0	0
12	NNov	Golan H	L5	12	Mixed	Mare	Pasture	6.5.15	37.5	Pink	1	17	0	5	2	0	10	30	8.2	1	1	1	0
12	NNov	Golan H	L6	5	Mixed	Mare	Pasture	6.5.15	36.6	Pink	1	28	0	22	3	0	3	33	8	1	1	1	0

12	NNov	Golan H	L7	9	Mixed	Gelding	Pasture	6.5.15	37	Pink	1	7	0	5	2	0	0	33	8.2	0	1	0	0
12	NNov	Golan H	L9	6	Mixed	Mare	Pasture	6.5.15	37.2	Pink	1	44	0	16	11	0	17	36	7.6	1	1	0	0
13	Merom Golan	Golan H	M1	12	Mixed	Mare	Pasture	6.5.15	36.2	Pink	1	23	0	12	6	0	5	34	7.2	1	1	0	1
13	Merom Golan	Golan H	M3	7	Mixed	Mare	Pasture	6.5.15	37.1	Pale	1	50	0	32	8	0	10	37	8.2	1	1	0	0
13	Merom Golan	Golan H	M4	18	Mixed	Mare	Pasture	6.5.15	37.8	Pink	1	44	4	26	3	0	11	38	7.2	1	1	0	0
13	Merom Golan	Golan H	M5	8	Mixed	Gelding	Pasture	6.5.15	37.4	Pale	1	27	0	16	4	0	7	34	7.2	1	1	1	0
13	Merom Golan	Golan H	M6	12	Mixed	Mare	Pasture	6.5.15	37.2	Pink	1	23	0	13	3	0	7	33	7.2	1	1	1	0
13	Merom Golan	Golan H	M8	12	Mixed	Mare	Pasture	6.5.15	38.1	Pink	1	12	0	7	4	0	1	32	7.6	1	1	0	0
13	Merom Golan	Golan H	M9	8	Mixed	Mare	Pasture	6.5.15	36.5	Pink	1	64	0	46	4	0	14	35	7.6	1	1	0	0
13	Merom Golan	Golan H	M10	8	Mixed	Mare	Pasture	6.5.15	37	Pink	1	44	0	29	9	0	6	34	7.4	1	1	0	0
13	Merom Golan	Golan H	M11	7	Mixed	Mare	Pasture	6.5.15	37.6	Pink	1	46	0	33	9	0	4	32	8	1	1	0	0
13	Merom Golan	Golan H	M12	7	Mixed	Mare	Pasture	6.5.15	37.6	Pink	1	33	0	18	7	0	8	36	7.2	1	1	1	0
13	Merom Golan	Golan H	M13	7	Mixed	Mare	Pasture	6.5.15	37	Pink	1	38	0	18	11	0	9	27	8	1	1	0	0
13	Merom Golan	Golan H	M14	14	Mixed	Gelding	Pasture	6.5.15	36.9	Pale	1	34	15	9	2	0	8	34	8	1	1	1	0
13	Merom Golan	Golan H	M15	10	Mixed	Gelding	Pasture	6.5.15	37	Pink	1	30	0	21	3	0	6	32	7.4	1	1	1	0
13	Merom Golan	Golan H	M16	18	Mixed	Mare	Pasture	6.5.15	36.9	Pale	1	22	0	13	5	0	4	36	7.6	1	1	1	0
13	Merom Golan	Golan H	M17	10	Mixed	Mare	Pasture	6.5.15	37.4	Pink	1	59	0	36	15	0	8	35	7.2	1	1	1	0
13	Merom Golan	Golan H	M18	14	Mixed	Gelding	Pasture	6.5.15	37	Pale	1	39	3	27	9	0	0	35	7.6	1	1	1	0
13	Merom Golan	Golan H	M19	4	Mixed	Mare	Pasture	6.5.15	37.2	Pale	1	49	0	38	5	0	5	35	7	1	1	1	1
13	Merom Golan	Golan H	M20	14	Mixed	Mare	Pasture	6.5.15	36	Pale	1	58	1	36	8	0	13	41	8.6	1	1	1	0
13	Merom Golan	Golan H	M21	6	Mixed	Gelding	Pasture	6.5.15	37.6	Pale	1	40	0	23	7	0	10	36	7.6	1	1	1	0
13	Merom Golan	Golan H	M23	14	Mixed	Mare	Pasture	6.5.15	37.6	Pale	1	42	0	26	6	0	10	40	7.6	0	1	1	0
13	Merom Golan	Golan H	M24	6	Mixed	Gelding	Pasture	6.5.15	37.5	Pink	1	31	0	17	0	0	14	35	7.6	1	1	1	0
13	Merom Golan	Golan H	M25	10	Mixed	Mare	Pasture	6.5.15	37.8	Pale	1	16	0	12	4	0	0	38	7	1	1	1	0
13	Merom Golan	Golan H	M26	7	Mixed	Mare	Pasture	6.5.15	37.6	Pink	1	21	1	14	1	0	5	35	7.2	1	1	1	0
13	Merom Golan	Golan H	M27	5	Mixed	Mare	Pasture	6.5.15	37.2	Pink	1	44	0	27	8	0	9	35	7.2	1	1	1	0
13	Merom Golan	Golan H	M28	12	Mixed	Gelding	Pasture	6.5.15	37.1	Pink	1	31	0	15	10	0	6	34	7.2	0	1	1	0
13	Merom Golan	Golan H	M29	4	Mixed	Gelding	Pasture	6.5.15	37.3	Pink	1	28	0	26	0	0	2	33	7.8	1	1	1	0
14	Ein Harod	North	N1	16	Mixed	Mare	Paddock	17.5.15	37.7	Pink	0	0	0	0	0	0	0	30	6.2	0	0	0	1
14	Ein Harod	North	N2	14	Mixed	Mare	Paddock	17.5.15	37.8	Pink	0	0	0	0	0	0	0	32	6.4	0	0	0	0
14	Ein Harod	North	N4	30	Mixed	Gelding	Paddock	17.5.15	37.2	Pink	0	0	0	0	0	0	0	31	6.4	1	1	0	0
14	Ein Harod	North	N6	5	Mixed	Mare	Paddock	17.5.15	37.6	Pink	0	0	0	0	0	0	0	34	6.4	1	1	0	0
14	Ein Harod	North	N7	11	Mixed	Mare	Paddock	17.5.15	37.6	Pink	0	0	0	0	0	0	0	35	7.2	0	0	0	0
14	Ein Harod	North	N8	17	Mixed	Mare	Paddock	17.5.15	38.3	Pink	0	0	0	0	0	0	0	33	7	0	0	1	0
14	Ein Harod	North	N10	18	Mixed	Mare	Paddock	17.5.15	37.3	Pink	0	0	0	0	0	0	0	34	6.4	0	0	0	0
15	Grufit	South	O1	21	Mixed	Mare	Paddock	27.5.15	37.6	Pink	0	0	0	0	0	0	0	33	7.4	0	0	0	0
15	Grufit	South	O2	15	Mixed	Mare	Paddock	27.5.15	37.1	Pale	0	0	0	0	0	0	0	35	7.4	1	1	1	0
15	Grufit	South	O3	20	Mixed	Gelding	Paddock	27.5.15	37.2	Pink	0	0	0	0	0	0	0	38	7.6	0	0	0	1
15	Grufit	South	O4	11	Quarter	Mare	Paddock	27.5.15	37.2	Pink	0	0	0	0	0	0	0	35	7	0	0	0	0
15	Grufit	South	O6	6	Mixed	Mare	Paddock	27.5.15	37	Pink	0	0	0	0	0	0	0	39	7.4	0	0	0	0
15	Grufit	South	O7	9	Mixed	Gelding	Paddock	27.5.15	37.6	Pink	0	0	0	0	0	0	0	38	7	0	0	1	0

15	Grufit	South	O8	7	Mixed	Gelding	Paddock	27.5.15	37.5	Pink	0	0	0	0	0	0	0	34	7.4	0	0	1	0
15	Grufit	South	O9	30	Mixed	Mare	Paddock	27.5.15	37.8	Pink	0	0	0	0	0	0	0	27	7.6	0	0	0	0
15	Grufit	South	O10	16	Mixed	Gelding	Paddock	27.5.15	37.3	Pink	0	0	0	0	0	0	0	33	6.8	0	0	1	0
15	Grufit	South	O11	22	Mixed	Gelding	Paddock	27.5.15	37.4	Pink	0	0	0	0	0	0	0	34	7.4	0	0	1	0
15	Grufit	South	O12	17	Mixed	Mare	Paddock	27.5.15	37.2	Pink	0	0	0	0	0	0	0	30	7.2	0	0	0	0
15	Grufit	South	O13	7	Mixed	Mare	Paddock	27.5.15	37.7	Pink	0	0	0	0	0	0	0	32	7.2	0	1	1	0
15	Grufit	South	O14	14	Quarter	Gelding	Paddock	27.5.15	37.9	Pink	0	0	0	0	0	0	0	32	6.6	0	0	1	0
15	Grufit	South	O15	14	Quarter	Gelding	Paddock	27.5.15	37.1	Pink	0	0	0	0	0	0	0	34	7	0	1	1	0
15	Grufit	South	O16	10	Mixed	Gelding	Paddock	27.5.15	37.9	Pink	0	0	0	0	0	0	0	32	6.6	0	0	1	0
15	Grufit	South	O17	5	Mixed	Mare	Paddock	27.5.15	38.3	Pink	0	0	0	0	0	0	0	26	7.2	0	0	1	0
16	Paran	South	P1	10	Quarter	Gelding	Paddock	27.5.15	37.8	Pink	0	0	0	0	0	0	0	34	6.4	0	0	0	0
16	Paran	South	P2	12	Quarter	Mare	Paddock	27.5.15	37.8	Pink	0	0	0	0	0	0	0	38	7	0	0	0	0
16	Paran	South	P3	10	Quarter	Gelding	Paddock	27.5.15	37.4	Pink	0	0	0	0	0	0	0	39	6.8	0	1	1	0
16	Paran	South	P4	0.75	Quarter	Mare	Paddock	27.5.15	38.6	Pink	0	0	0	0	0	0	0	33	5.8	0	0	0	0
18	Vered Hagalil	North	R1	12	Pony	Mare	Pasture	2.6.15	37.4	Pink	0	0	0	0	0	0	0	38	6.8	1	1	1	0
18	Vered Hagalil	North	R2	8	Mixed	Mare	Pasture	2.6.15	37	Pink	0	0	0	0	0	0	0	37	7.2	1	1	1	0
18	Vered Hagalil	North	R3	10	Mixed	Mare	Pasture	2.6.15	37.2	Pink	0	0	0	0	0	0	0	37	7	1	1	1	0
18	Vered Hagalil	North	R4	8	Mixed	Gelding	Pasture	2.6.15	37.1	Pink	1	1	0	1	0	0	0	35	7.2	1	1	1	0
18	Vered Hagalil	North	R5	28	Mixed	Gelding	Pasture	2.6.15	37.6	Pink	0	0	0	0	0	0	0	36	7.2	1	1	1	0
18	Vered Hagalil	North	R7	8	Mixed	Gelding	Pasture	2.6.15	38.2	Pink	0	0	0	0	0	0	0	38	6.8	1	1	1	0
18	Vered Hagalil	North	R8	10	Mixed	Gelding	Pasture	2.6.15	37.1	Pink	0	0	0	0	0	0	0	30	7.2	1	1	0	0
18	Vered Hagalil	North	R9	9	Quarter	Gelding	Pasture	2.6.15	37.8	Pink	1	1	0	1	0	0	0	32	6	1	1	1	0
18	Vered Hagalil	North	R10	10	Quarter	Gelding	Pasture	2.6.15	37.1	Pink	1	1	0	0	1	0	0	26	6.4	1	1	1	0
18	Vered Hagalil	North	R11	8	Mixed	Gelding	Pasture	2.6.15	37.4	Pink	1	1	0	1	0	0	0	31	7.4	1	1	1	0
18	Vered Hagalil	North	R12	6	Mixed	Mare	Pasture	2.6.15	37.2	Pink	0	0	0	0	0	0	0	30	7.4	1	1	0	0
18	Vered Hagalil	North	R13	6	Mixed	Gelding	Pasture	2.6.15	37.3	Pink	1	2	0	2	0	0	0	28	6.6	1	1	1	0
18	Vered Hagalil	North	R14	10	Quarter	Mare	Pasture	2.6.15	37.8	Pink	0	0	0	0	0	0	0	36	7.4	1	1	1	0
18	Vered Hagalil	North	R15	12	Quarter	Mare	Pasture	2.6.15	37.1	Pink	0	0	0	0	0	0	0	32	7.4	1	1	1	0
18	Vered Hagalil	North	R16	10	Quarter	Gelding	Pasture	2.6.15	37.3	Pink	0	0	0	0	0	0	0	31	6.6	1	1	0	0
18	Vered Hagalil	North	R17	6	Mixed	Gelding	Pasture	2.6.15	36.9	Pink	0	0	0	0	0	0	0	38	7.2	1	1	1	0
18	Vered Hagalil	North	R18	20	Mixed	Gelding	Pasture	2.6.15	37	Pale	0	0	0	0	0	0	0	32	8	1	1	1	0
19	Iblin	North	S1	7	Arabian	Stallion	Stall	17.5.15	36	Pink	0	0	0	0	0	0	0	44	6.6	0	0	1	0
19	Iblin	North	S2	5	Arabian	Mare	Stall	17.5.15	36.5	Pink	0	0	0	0	0	0	0	42	6.8	0	0	1	0
19	Iblin	North	S3	3	Arabian	Mare	Stall	17.5.15	37.1	Pink	0	0	0	0	0	0	0	33	7.8	0	0	1	0
19	Iblin	North	S4	4	Arabian	Stallion	Stall	17.5.15	37.8	Pink	0	0	0	0	0	0	0	49	6.8	0	0	0	0
19	Iblin	North	S5	5	Arabian	Mare	Stall	17.5.15	37.6	Pink	0	0	0	0	0	0	0	42	7.6	0	0	0	0
19	Iblin	North	S6	1.5	Arabian	Mare	Stall	17.5.15	37.1	Pink	0	0	0	0	0	0	0	47	7.4	0	0	0	0
19	Iblin	North	S8	7	Arabian	Mare	Stall	17.5.15	37.2	Pink	0	0	0	0	0	0	0	46	7	0	0	1	0
19	Iblin	North	S9	3	Arabian	Mare	Stall	17.5.15	37.1	Pink	0	0	0	0	0	0	0	42	7	0	0	0	0
20	Mevo Hama	Golan H	T1	4	Mixed	Gelding	Pasture	6.5.15	37.7	Pink	1	26	0	16	0	0	10	32	7.2	1	1	1	0
20	Mevo Hama	Golan H	T2	4	Mixed	Gelding	Pasture	6.5.15	37.7	Pale	1	14	0	12	0	0	2	35	7.6	1	1	1	1

20	Mevo Hama	Golan H	T3	3	Mixed	Gelding	Pasture	6.5.15	37.4	Pink	1	12	0	4	0	0	8	35	8.2	1	1	1	0
20	Mevo Hama	Golan H	T4	12	Mixed	Mare	Pasture	6.5.15	37.6	Pink	1	40	0	12	0	0	28	33	9	1	1	1	0
20	Mevo Hama	Golan H	T5	3	Mixed	Mare	Pasture	6.5.15	38	Pink	1	41	0	15	0	1	25	33	8.6	1	1	1	0
20	Mevo Hama	Golan H	T6	4	Mixed	Gelding	Pasture	6.5.15	37.5	Pink	1	3	0	3	0	0	0	33	8	1	1	1	0
20	Mevo Hama	Golan H	T7	6	Mixed	Mare	Pasture	6.5.15	37.1	Pink	1	28	0	25	0	0	3	33	8	1	1	1	1
20	Mevo Hama	Golan H	T8	6	Mixed	Gelding	Pasture	6.5.15	37	Pink	1	16	0	12	0	0	4	32	7.4	1	1	1	0
20	Mevo Hama	Golan H	T9	5	Mixed	Gelding	Pasture	6.5.15	36.5	Pink	1	12	0	4	0	0	8	36	7	1	1	1	1
21	Urim	South	U1	13	Mixed	Mare	Stall	15.6.15	37.3	Pink	1	1	0	1	0	0	0	28	6.2	0	1	0	0
21	Urim	South	U2	20	Mixed	Mare	Stall	15.6.15	36	Pink	0	0	0	0	0	0	0	37	6.6	1	1	1	0
21	Urim	South	U3	15	Mixed	Mare	Stall	15.6.15	37.1	Pale	0	0	0	0	0	0	0	27	6.4	0	0	0	0
21	Urim	South	U4	9	Mixed	Mare	Pasture	15.6.15	37.4	Pink	0	0	0	0	0	0	0	27	6.4	1	1	0	0
21	Urim	South	U6	6	Tennessee	Gelding	Pasture	15.6.15	37	Pale	1	1	0	1	0	0	0	28	6.4	0	0	0	0
21	Urim	South	U7	13	Mixed	Mare	Stall	15.6.15	36.7	Pink	0	0	0	0	0	0	0	34	6.4	0	0	0	0
